# Pulsed electric field-mediated elimination of yeast [*PSI*^+^] prion is variant dependent and affects cell growth dynamics

**DOI:** 10.7717/peerj.21775

**Published:** 2026-10-23

**Authors:** Neda Jonutyte-Trembo, Justina Versockienė, Gvidas Katauskas, Vitalij Novickij, Egle Lastauskiene

**Affiliations:** 1 Institute of Biosciences, Life Sciences Center, Vilnius University, Vilnius, Lithuania; 2 Faculty of Electronics, Vilnius Gediminas Technical University, Vilnius, Lithuania

**Keywords:** PSI prion, Yeast prions, Prion curing, Pulsed electric field, Growth dynamics

## Abstract

Yeast *Saccharomyces cerevisiae* is a widespread model system in which mechanisms of prion induction and elimination have been identified. New and safe substances and methods are being sought to eliminate prions from cells. In this research, we applied guanidinium hydrochloride (GuHCl) alone and in combination with pulsed electric field treatment against [*PSI*^+^] prion. The analysis of cell culture optical density and doubling time showed that the method of [*PSI*^+^] prion elimination increases cell doubling time, with combined treatment having a more severe impact on cell growth than GuHCl treatment alone. Additionally, we found that both elimination methods can impact the optical culture density achieved by [*psi*^−^] cells. However, the cells in which the [*PSI*^+^] prion has been eliminated by GuHCl alone are able to reach the same optical density as unaffected [*psi*^−^] cells and a higher optical density than the affected [*psi*^−^] cells by GuHCl alone. These findings suggest that the presence and subsequent removal of the [*PSI*^+^] prion influence early exponential growth, revealing lasting effects on cell growth dynamics that are distinct from previously reported effects on stationary phase survival. This work highlights how prion elimination strategies, including chemical and electric field treatments, can impact post-curing growth behavior in yeast cells.

## Introduction

Prions are proteinaceous infectious particles that act as pathogens and cause the development of lethal neurodegenerative diseases in humans and other animals (Prusiner, 1982). Prions, unlike other pathogens such as bacteria, viruses, or fungi, are composed of proteins and do not contain nucleic acids. These proteins tend to aggregate and are particularly resistant to a variety of physical, chemical, and biological agents (Hunter & Millson, 1964; Pattison, 1965; Hunter et al., 1969). Due to these unique properties of prions, they are actively studied. Prion protein studies in humans or other mammals are hampered by long lifespan, ethical constraints, and other issues. Therefore, model systems of lower eukaryotes are often used. Yeast *Saccharomyces cerevisiae* is widely used as a model system for studying protein structures, functions, and interactions and can be compared to higher eukaryotic systems due to the high conservativity of biological mechanisms (Castrillo & Oliver, 2011). Mechanisms of prion induction, propagation, aggregate formation, elimination, and interactions of different prion proteins have been identified in the yeast model system (Wickner et al., 2016).

At least 10 prion proteins have been identified in *S. cerevisiae* cells (Wickner, 1994; Du et al., 2008; Alberti et al., 2009; Parfenova & Barral, 2020), including the [*PSI* +] prion, which has been known for more than two decades (Wickner, 1994) and is still under intense research. The altered structure of the Sup35 protein leads to protein aggregation and the emergence of the [*PSI*^+^] phenotype (King et al., 1997; Serio et al., 2000; Tuite, 2013). The native Sup35 protein, also known as eRF3, is involved in translation termination (Zhouravleva et al., 1995). When cells are in the [*PSI*^+^] state, Sup35 proteins are aggregated, insoluble, and thus non-functional. This results in a decrease in the efficacy of the translation termination (Paushkin et al., 1996; Patino et al., 1996).

Different prion states were identified in both mammalian and yeast cells and were named prion variants. These variants may form distinct phenotypes that differ in prion protein stability and frequency of spontaneous occurrence in mammals, as well as prion protein and mitotic stability and elimination efficiency in yeast. In the yeast system, different variants of [*PSI*^+^] prion are called weak and strong (Derkatch et al., 1996), which differ in the ratio of the aggregated Sup35 and the efficiency of translation termination. The weak [*PSI*^+^] prion variant has a smaller proportion of aggregated Sup35 protein, whereas the strong [*PSI*^+^] prion variant has a larger proportion of aggregated Sup35 protein (Zhou, 1999; Uptain, 2001). It is increasingly important to distinguish different prion variants and their effect on the cell because they can cause pathologically distinct infectious diseases in animals (Xiao et al., 2020).

The significance of the appearance of the [*PSI*^+^] prion is debatable. Some researchers suggest that prions may be beneficial to yeast under adverse environmental conditions because they help cells adapt and survive (Eaglestone, 1999; True & Lindquist, 2000; Tyedmers, Madariaga & Lindquist, 2008). Others argue that prions do not provide survival benefits (Wickner et al., 2011) because, in wild-type yeast cells, prions are relatively rare (Chernoff et al., 2000; Nakayashiki et al., 2005). It has been shown that the [*PSI*^+^] prion prolongs the chronological lifespan of yeast cells. As cells grow for an extended period, nutrients become depleted, and growth conditions become increasingly challenging (True & Lindquist, 2000; Tyedmers, Madariaga & Lindquist, 2008; Wang, Melki & Kabani, 2017). Under such stressful conditions, [*PSI*^+^] prions help cells adapt and survive longer, even after [*PSI*^+^] curing. Either way, prions cause lethal diseases in mammals; therefore, research on prion elimination is being carried out intensively (Chernova, Chernoff & Wilkinson, 2019).

New and safe substances and methods are being sought to eliminate prion proteins from cells (Glover & Lindquist, 1998). The goal of most of these studies is to achieve the highest possible cure efficiency and the lowest probability of recurrence of the prion protein. Guanidine hydrochloride (GuHCl) is a chemical compound commonly used to eliminate prions in yeast cells. At low concentrations, GuHCl attenuates the ATPase activity of the Hsp104 chaperone, thereby reducing fibril fragmentation and preventing prion inheritance (Glover & Lindquist, 1998; Jung & Masison, 2001). Hsp104 is essential for the fragmentation and proliferation of amyloid fibrils, while decreased activity of this protein results in the formation of long fibrils that can no longer enter the daughter cells (True & Lindquist, 2000). In addition to prion elimination, physical factors such as the pulsed electric field (PEF) can be used (Jurgelevičiūtė et al., 2021). Prion proteins are highly resistant to traditional protein denaturation conditions, such as proteases, heat, and acids (Linden et al., 2008), and their formation is strongly influenced by electrostatic interactions (Guest, Cashman & Plotkin, 2010). Recent work has shown that PEF can shorten Sup35NM fibrils and modify prion stability in yeast (Jurgelevičiūtė et al., 2021). PEF-induced changes in fibril structure could have opposing effects on prion maintenance. Extensive aggregate disruption could favor elimination, whereas fibril shortening could preserve or increase the number of aggregation-competent particles. The latter possibility remains hypothetical because propagon number and seeding activity after PEF treatment have not been measured directly. Responses may also differ between [*PSI*^+^] variants because strong- and weak-variant Sup35 fibrils exhibit distinct morphologies (Ghosh et al., 2018). These observations provide a mechanistic rationale for testing whether PEF enhances or interferes with GuHCl-mediated [*PSI*^+^] curing in a variant-dependent manner.

Here, we demonstrate that eliminating [*PSI*^+^] prions with GuHCl in combination with PEF does not increase elimination efficiency and may cause long-term changes in the cell that affect its growth. We also show that the [*PSI*^+^] prion may be beneficial for early cell growth (in the absence of stressful conditions). Unlike previous studies that focused on [*PSI*^+^]-associated stationary phase survival (Wang, Melki & Kabani, 2017), this work specifically examines how different curing strategies influence early exponential growth and post-curing cell behavior, using both phenotypic and biochemical approaches.

## Materials & Methods

Portions of this text were previously published as part of a preprint (Versockienė et al., 2022). This study aims to determine the growth dynamics of *S. cerevisiae* cells carrying different [*PSI*^+^] prion variants, cells that have lost the prion, and cells that never contained [*PSI*^+^]. To achieve this goal, [*PSI*^+^] prion was induced in yeast cells, and weak and strong [*PSI*^+^] prion variants were isolated by established phenotypic assays. [*PSI*^+^] prion elimination was carried out using GuHCl alone or in combination with PEF treatment. Prion states (present, eliminated, absent) were verified using colony color on $ \frac{1}{4} $ YPD medium, growth on SC medium lacking adenine, and Western blot analysis of Sup35 aggregates. All prionized, cured, and prion-free control strains were grown for 48 h, and their optical density and doubling time were measured to evaluate early exponential growth behavior.

### Yeast strains

*S. cerevisiae* 74-D694 strain (*MAT****a*,** *ade1-14 (UGA), trp1-289, his3-*Δ*200, ura3-52, leu2-3,112* [*PIN*^+^][*psi*^−^]) was used in our experiments (Chernoff et al., 1995). This strain is auxotrophic for adenine, tryptophan, histidine, uracil, and leucine, which must be provided in the growth medium. *ade1* mutant phenotypes allow the discrimination of the cells without prions or containing strong or weak prions by the color of the colonies (Fig. 1). In addition to colony color on $ \frac{1}{4} $ YPD, selected isolates were also planted on synthetic medium lacking adenine (SC-Ade) to confirm the nonsense-suppression phenotype characteristic of [*PSI*^+^] cells. SC-ade plates showed the expected growth patterns: [*PSI*^+^] variants grew due to stop codon readthrough, whereas [*psi*^−^] cells failed to grow. Strains used in this work are listed in Table 1.

**Figure 1:**
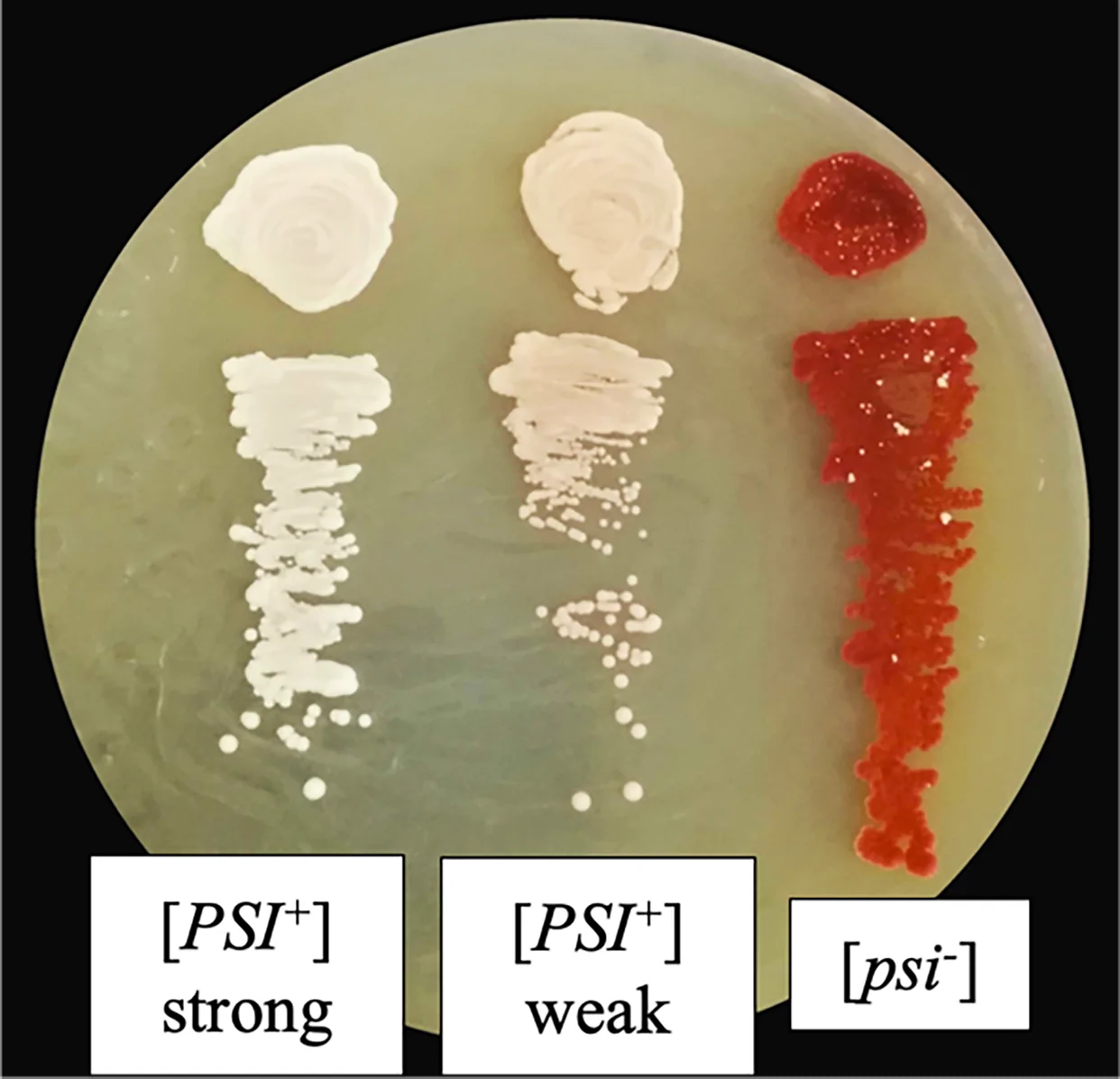
Phenotype of *ade1* mutants on YPD medium. White cells containing strong [*PSI*^+^] prion, pink cells containing weak [*PSI*^+^] prion, and red cells without [*PSI*^+^] prion ([*psi*^−^]).

**Table 1:** *S. cerevisiae* 74-D694 strains used in this work.

Abbreviation	Phenotype	Source
[*psi*^−^]	*S. cerevisiae* 74-D694 *MATa, ade1-14 (UGA), trp1-289, his3-*Δ*200, ura3-52, leu2-3,112* [*psi*^−^][*PIN*^+^]	Gift from prof. Yury Chernoff
[*PSI*^+^]-w	*S. cerevisiae* 74-D694 [*PSI*^+^] weak	This work
[*PSI*^+^]-s	*S. cerevisiae* 74-D694 [*PSI*^+^][*PIN*^+^] strong	This work
[*psi*^−^]-G	*S. cerevisiae* 74-D694 [*psi*^−^][*pin*^−^] treated with GuHCl	This work
[*psi*^−^]-GP	*S. cerevisiae* 74-D694 [*psi*^−^][*pin*^−^] treated with GuHCl and PEF	This work
[*psi*^−^]-w-G	*S. cerevisiae* 74-D694 [*psi*^−^][*pin*^−^] weak cured with GuHCl	This work
[*psi*^−^]-w-GP	*S. cured* 74-D694 [*psi*^−^][*pin*^−^] weak eliminated with GuHCl and PEF	This work
[*psi*^−^]-s-G	*S. cerevisiae* 74-D694 [*psi*^−^][*pin*^−^] strong eliminated with GuHCl	This work
[PSI^+^]-s-GP	*S. cerevisiae* 74-D694 [*PSI*^+^][*PIN*^+^] strong not eliminated with GuHCl and PEF treatment	This work

### Spontaneous prionisation and selection of different prion variants

*S. cerevisiae* [*psi*^−^] cells were plated on an agarized YPD medium (2% glucose, 2% peptone, 1% yeast extract, 2% agar) and grown for 72 h at 30 °C. One colony was then transferred to five mL of fresh YPD medium and grown overnight (∼12 h) at 30 °C with aeration (130 rpm). Overnight culture was washed with sterile distilled water, and approximately 10^7^ cells were plated on an agarized SC medium lacking adenine (SC-Ade; 2% glucose, 0.67% yeast nitrogen base without amino acids, 2% agar, supplemented according to strain genotype but without adenine and grown for 21 days at 30 °C (Fig. S1). Colonies appearing on SC-Ade were transferred to plates containing agarized $ \frac{1}{4} $ YPD medium (the same as YPD, but with 0.25% yeast extract) and analyzed for colony color. White colonies were classified as strong [*PSI*^+^] ([*PSI*^+^]-s), and pink colonies as weak [*PSI*^+^] ([*PSI*^+^]-w). A subset of isolates was re-plated onto SC-Ade to confirm the nonsense suppression phenotype typical of [*PSI*^+^] cells. Cells cured with guanidine hydrochloride and plated on SC-Ade media to confirm no revertant mutation has occurred in *ade1* gene. One representative isolate of each variant was selected for subsequent experiments.

### Prion elimination (“curing”)

*S. cerevisiae* [*psi*^−^], [*PSI*^+^]-w, and [*PSI*^+^]-s cells were plated on agarized YPD medium and grown for 48–72 h at 30 °C. One colony of the respective culture was then transferred to five mL of fresh YPD medium and grown overnight (∼12 h) at 30 °C with aeration (130 rpm). The overnight culture was transferred to 50 mL of fresh YPD medium containing five mM GuHCl to a final optic density (OD) of 0.06 AU at 600 nm and grown for 35 h, at 30 °C with aeration. A portion of each culture was subjected to pulsed electric field (PEF) treatment at 10 kV/cm (five pulses of one ms each). Pulses of one kV were applied in a one mm gap aluminium cuvette (Bio-Rad, Hercules, CA, USA), producing a 10 kV/cm electric field; the parameters were selected based on previously performed viability tests (Jurgelevičiūtė et al., 2021). After treatment, all cultures (GuHCl-treated, GuHCl + PEF-treated, and untreated controls) were plated on YPD for 72 h and then replica-plated onto SC-Ade medium for 72 h. Only red color colonies on YPD medium (Fig. S2) that failed to grow on SC-Ade were considered. These yielded the following cured phenotypes:

1) [*psi*^−^] cured with GuHCl ([*psi* ^−^]-G);

2) [*psi*^−^] cured with GuHCl and PEF ([*psi*^−^]-GP);

3) [*PSI*^+^] weak cured with GuHCl ([*psi* ^−^]-w-G);

4) [*PSI*^+^] weak cured with GuHCl and PEF ([*psi*^−^]-w-GP);

5) [*PSI*^+^] strong cured with GuHCl ([*psi* ^−^]-s-G);

Prion elimination efficiency was calculated as the percentage of [*psi*^−^] colonies relative to all colonies grown.

### Cell growth dynamics

*S. cerevisiae* strains listed in Table 1 were plated on agarized YPD medium and grown for 48 h at 30 °C. One colony of each culture was then transferred to five mL of fresh YPD medium and grown overnight (∼12 h) at 30 °C with aeration (130 rpm). Overnight cultures were diluted into 50 mL of fresh YPD medium to a final OD of 0.06 AU at 600 nm. Culture samples were taken every 3 h and cell growth was measured using a biophotometer (Eppendorf, Hamburg, Germany). Growth dynamics were observed until the culture reached the stationary growth phase. Exponential growth phase was defined as between 3 and 12 h after initial inoculation. After growth measurements, cells were plated onto agarized $ \frac{1}{4} $ YPD and SC-Ade media to verify that their [*PSI*^+^]/[*psi*^−^] status had not changed during the experiment. All the experiments were performed in three independent biological replicates.

### Growth rate and doubling time

The growth rate (*gr*) was calculated with Eq. (1):

(1) \begin{eqnarray*}gr= \frac{\ln \nolimits \left( \frac{N(t)}{N(0)} \right) }{t} \end{eqnarray*}

N(t)–number of cells at time t;

N(0)–number of cells at time 0;

t–time, h.

The doubling time (*DT*) was calculated with Eq. (2):

(2) \begin{eqnarray*}DT= \frac{\ln \nolimits (2)}{gr} \end{eqnarray*}

gr–growth rate.

Calculations performed according to Vandermeer (2010).

### Statistical analysis

At least three independent experimental sessions were performed for each set of conditions. Data normality was assessed using the Shapiro–Wilk test, and homogeneity of variance was evaluated using the *F*-test. A one-tailed *t*-test with Welch’s correction—or a nonparametric one-tailed Mann–Whitney U test, when the assumptions of the *t*-test were not met—was used only for the prespecified directional hypothesis that combined GuHCl + PEF treatment would increase prion-curing efficiency relative to GuHCl alone. No directional hypothesis was assumed for growth-related outcomes. Maximal optical density and cell doubling time were analyzed using Welch’s one-way analysis of variance (ANOVA). Pairwise comparisons were adjusted for multiple testing using the Benjamini–Hochberg false discovery rate procedure. Results were considered statistically significant at an adjusted *p*-value of <0.05. Statistical analysis was performed with GraphPad Prism (v 10.5.0). Raw data are presented in Supplementary file 1.

### SDS-PAGE, SDD-AGE, Western blot

Semi-denaturing agarose gel electrophoresis and immunoblotting were performed according to previous literature (Halfmann & Lindquist, 2008; Marchante et al., 2013). Briefly, *S. cerevisiae* strains listed in Table 1 were grown to log phase in YPD at 30 °C with aeration. Cells were collected and lysed using glass beads in the lysis buffer (100 mM Tris–HCl pH 7.5, 50 mM NaCl, 1 mM DTT, and 1:100 ThermoFisher protease inhibitor cocktail III; Thermo Fisher Scientific, Waltham, MA, USA). Protein concentration was measured by UV absorption. For SDS-PAGE, 20 µg of lysates were denaturated in 2x Laemmli buffer (2% SDS, 20% glycerol, 100 mM DTT, 0.125 M Tris–HCl, pH 6.8) 10 min at 95 °C, run on 4–20% Tris-glycine PAGE gel (Novex, Thermo Fisher Scientific) in Tris-glycine buffer (192 mM glycine, 25 mM Tris, 0.1% SDS).

For SDD-AGE, ∼100 µg of lysates were mixed with 2x sample buffer (2x TAE, 20% glycerol, 2% SDS), incubated for 10 min at room temperature and electrophoresed in a 1.7% agarose gel in running buffer (200 mM glycine, 20 mM Tris) with 0.1% SDS.

In both cases, proteins were transferred to 0.2 µm nitrocellulose membrane (Bio-Rad) using a semi-dry blotting apparatus with transfer buffer (200 mM glycine, 20 mM Tris, 15% v/v ethanol, 0.01% SDS), blocked in 5% skim milk solution, and probed with anti-Sup35 antibody (1:1000, #PAB15578; Abnova) and HRP-conjugated secondary antibody (1:10000, #PAB10822; Abnova). For loading control, blot from SDS-PAGE gel was stripped with stripping buffer (15 g/L glycine, 1 g/L SDS, 10 mL/L Tween 20, pH 2.2), reblocked, and probed for GAPDH with HRP-conjugated primary antibody (1:20000, #HRP-60004; Proteintech). Detection was done on Thermo Fisher FL1500 imager in automatic “Smart Exposure” mode (6x6 binning mode) with SuperSignal West PicoPlus substrate (Thermo Fisher Scientific).

Uncropped gel figures are shown in Fig. S3.

## Results

### Prion elimination efficiency

After prionisation, cell colonies containing weak and strong [*PSI*^+^] variants were isolated based on colony color on $ \frac{1}{4} $ YPD medium. The prion states of the isolated variants and their cured derivatives were further verified by growth on SC-Ade plates and by Western blot detection of Sup35 aggregates (Fig. 2A). Weak and strong [*PSI*^+^] variants were eliminated by GuHCl or GuHCl combined with PEF. The weak [*PSI*^+^] variant was eliminated more efficiently than the strong variant. When GuHCl alone was used, the weak [*PSI*^+^] prion was eliminated in 62.55 ± 8.52% of cells (Table S1.1), whereas the strong [*PSI*^+^] prion was eliminated in 35.63 ± 5.73% of cells (Fig. 3; Table S1.2). As shown below, the strong [*PSI*^+^] variant was not eliminated by combined GuHCl + PEF treatment.

**Figure 2:**
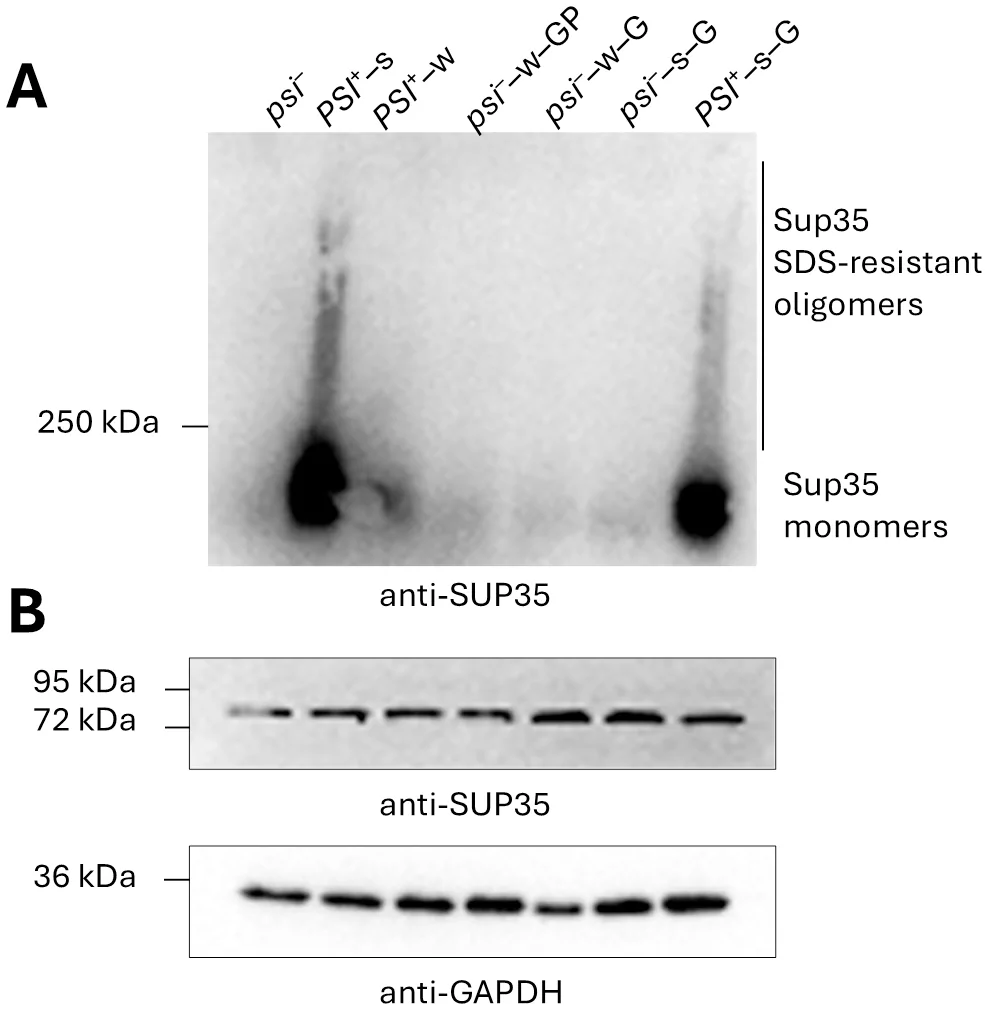
Immunoblot experiments confirming curing of [*PSI*^+^] prion. (A) Anti-Sup35 immunoblot after SDD-AGE experiment, showing no large SDS-resistant oligomers in cured strains, (B) same lysates were separated on SDS-PAGE gel and probed for Sup35 and GAPDH (as loading control), with no detectable change in Sup35 in cured strains. Sample abbrevations in the figures are: *psi*^−^–wildtype 74-D694 yeast strain, lacking [*PSI*^+^] prion, used in this study, *PSI*^+^-s–wildtype [*PSI*^+^] strong prion variant, *PSI*^+^-w –wildtype [*PSI*^+^] weak prion variant, *psi*^−^-w-G –[*PSI*^+^] weak cured with GuHCl, *psi*^−^-w-GP –[*PSI*^+^] weak cured with GuHCl and PEF, *psi*^−^-s-G –[*PSI*^+^] strong cured with GuHCl, *PSI*^+^ -s-GP –*PSI*^+^strong treated with GuHCl and PEF but not cured by treatment.

**Figure 3:**
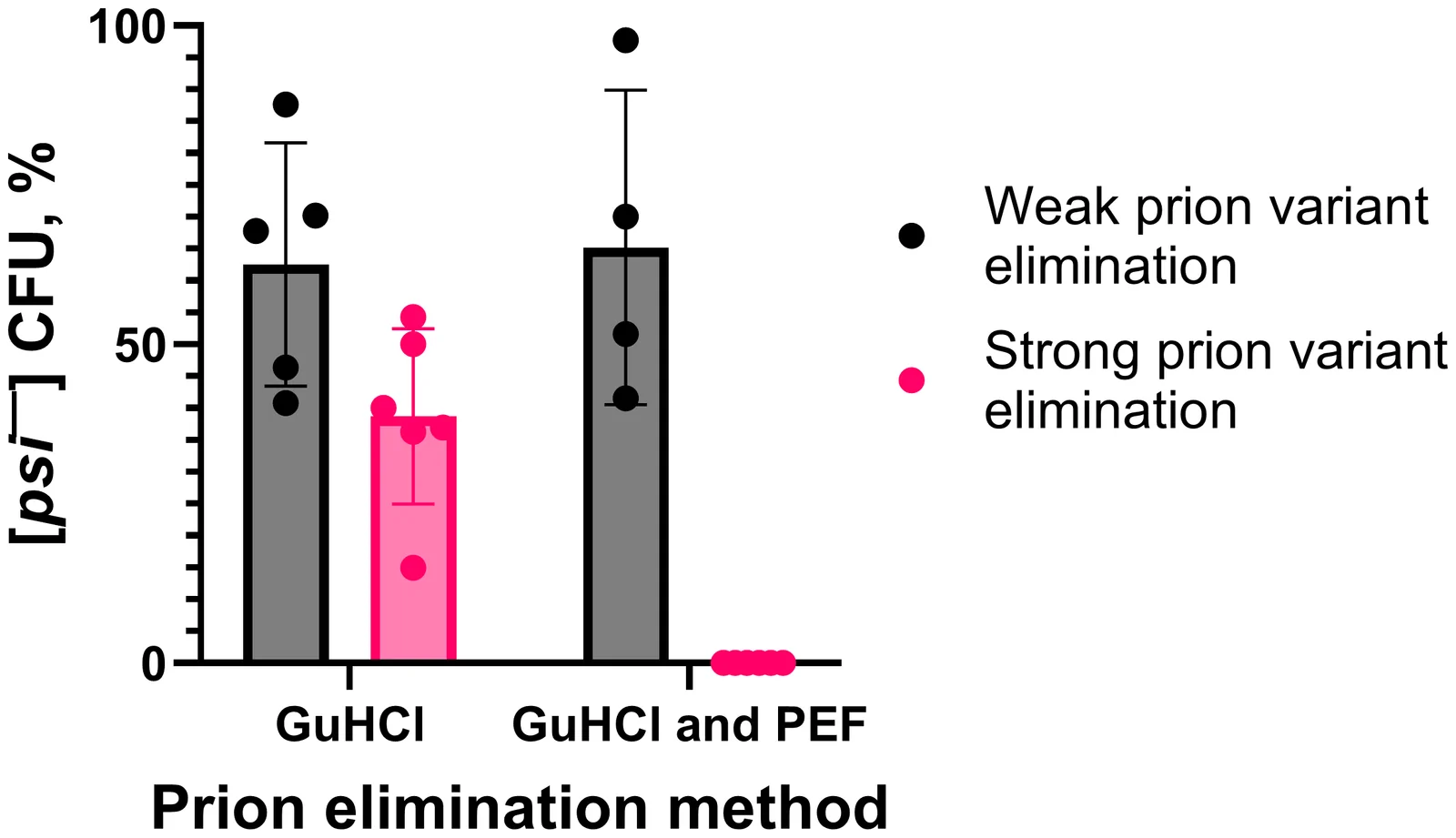
Percentage of the cells eliminated from the weak or strong [*PSI*^+^] prion variant. CFU, colonies forming units; GuHCl, guanidine hydrochloride; PEF, pulsed electric field. Strong [*PSI*^+^] prions could not be eliminated using GuHCl and PEF. Data presented as average ± SE.

SDD-AGE and immunoblot methods were used to confirm prion curing. Both weak and strong [*PSI*^+^] variants showed the expected high molecular weight Sup35 aggregate signal, which was absent in the [*psi*^−^] variant (Fig. 2A), but no change in overall Sup35 levels were detected (Fig. 2B). After GuHCl curing, Sup35 aggregates were no longer detected in [*psi*^−^]-w-G and [*psi*^−^]-s-G cells, consistent with efficient elimination of weak and strong variants. Additionally, [*psi*^−^]-w-GP cured from the weak prion variant by the combined GuHCl + PEF treatment also lacked detectable aggregates, whereas strong [*PSI*^+^] cells treated with GuHCl + PEF retained a robust Sup35 aggregate pattern similar to the untreated strong [*PSI*^+^] strain. These biochemical data corroborate the phenotypic assay results.

The weak [*PSI*^+^] prion variant was eliminated with similar efficiency using either GuHCl alone or the combination of GuHCl and PEF (65.2 ± 11.03%), and no statistically significant difference was observed between the two treatments (*p* > 0.05; Table S1.3). In contrast, the strong [*PSI*^+^] prion variant was eliminated only when GuHCl was used (33.63 ± 5.73%), while no elimination was detected following the combined GuHCl + PEF treatment (0%). This difference in elimination efficiency between the two treatments was statistically significant (*p* < 0.05; Table S1.4).

### Cell growth dynamics

A study of cell growth dynamics was conducted for cells containing the weak [*PSI*^+^] variant, cells cured of [*PSI*^+^] prion, and [*psi*^−^] cells that have never contained [*PSI*^+^]. The growth curves of these strains are shown in Fig. 4 (Tables S2.1–S2.3, S2.6).

**Figure 4:**
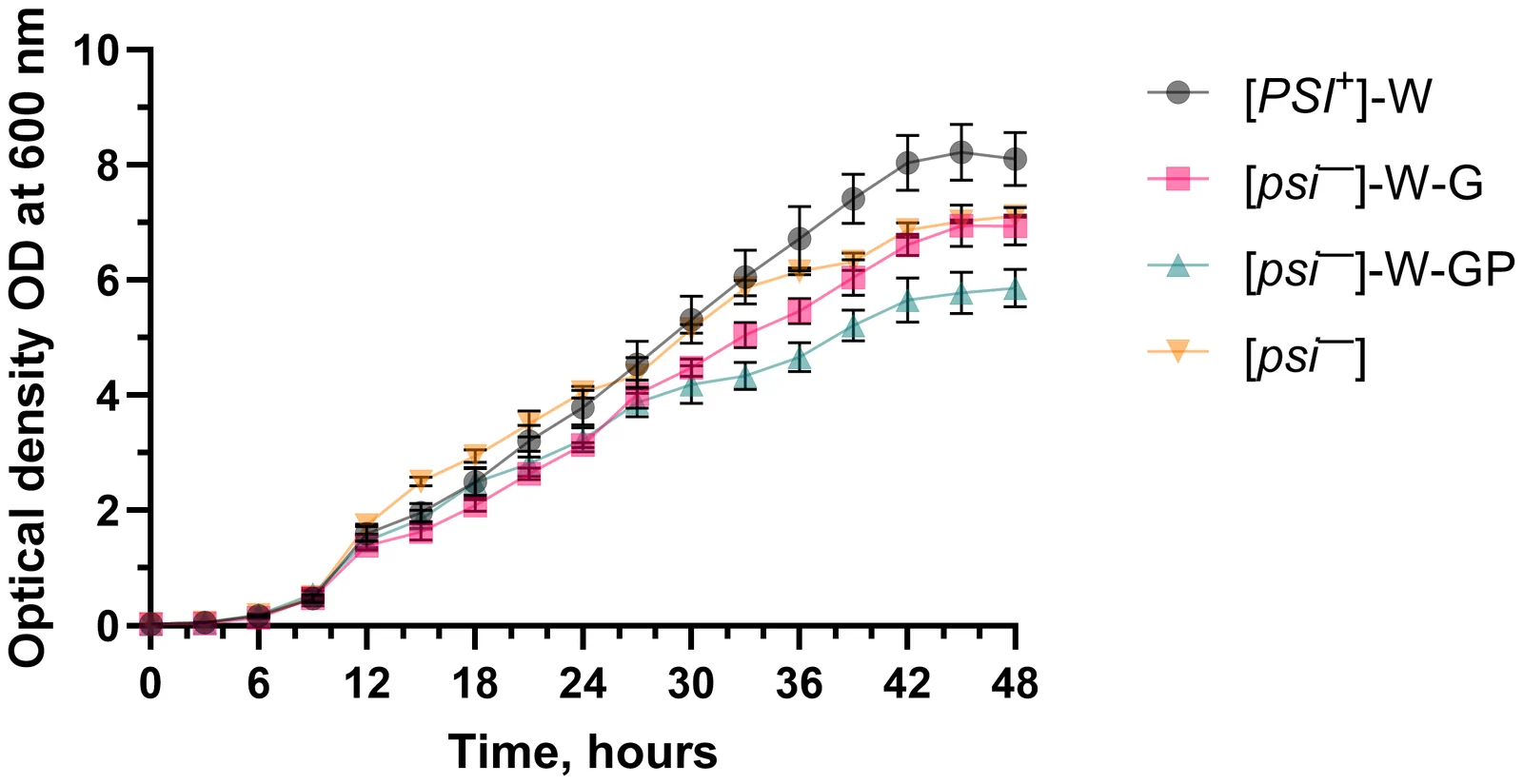
Growth dynamics of the cells with and without [*PSI*^+^] prion (weak variant). OD, optical density. Data presented as average ± SE.

The highest optical density was observed in cells containing the weak [*PSI*^+^] prion variant (8.22 ± 0.48 at 45 h). Neither cured cells nor [*psi*^−^] cells reached this level of biomass accumulation. Among the cured strains, cells in which the weak [*PSI*^+^] variant was eliminated using GuHCl alone reached a significantly higher OD (6.94 ± 0.32 at 48 h) than cells cured using the combined GuHCl + PEF method (5.87 ± 0.33 at 48 h; *p* < 0.05, Table S2.9). For the cells that have never had a [*PSI*^+^] prion, the highest reached OD was 7.11 ± 0.01 at 48 h, this value is similar to the cells in which the weak [*PSI*^+^] prion variant was eliminated using GuHCl alone (*p* > 0.05, Table S2.10). These results indicate that the weak [*PSI*^+^] prion variant enhances biomass accumulation and that the curing method influences the post-curing growth capacity of cells.

The growth of the cells with a strong [*PSI*^+^] prion variant and the cells eliminated from this variant is shown in Fig. 5 (Tables S2.4–S2.6).

**Figure 5:**
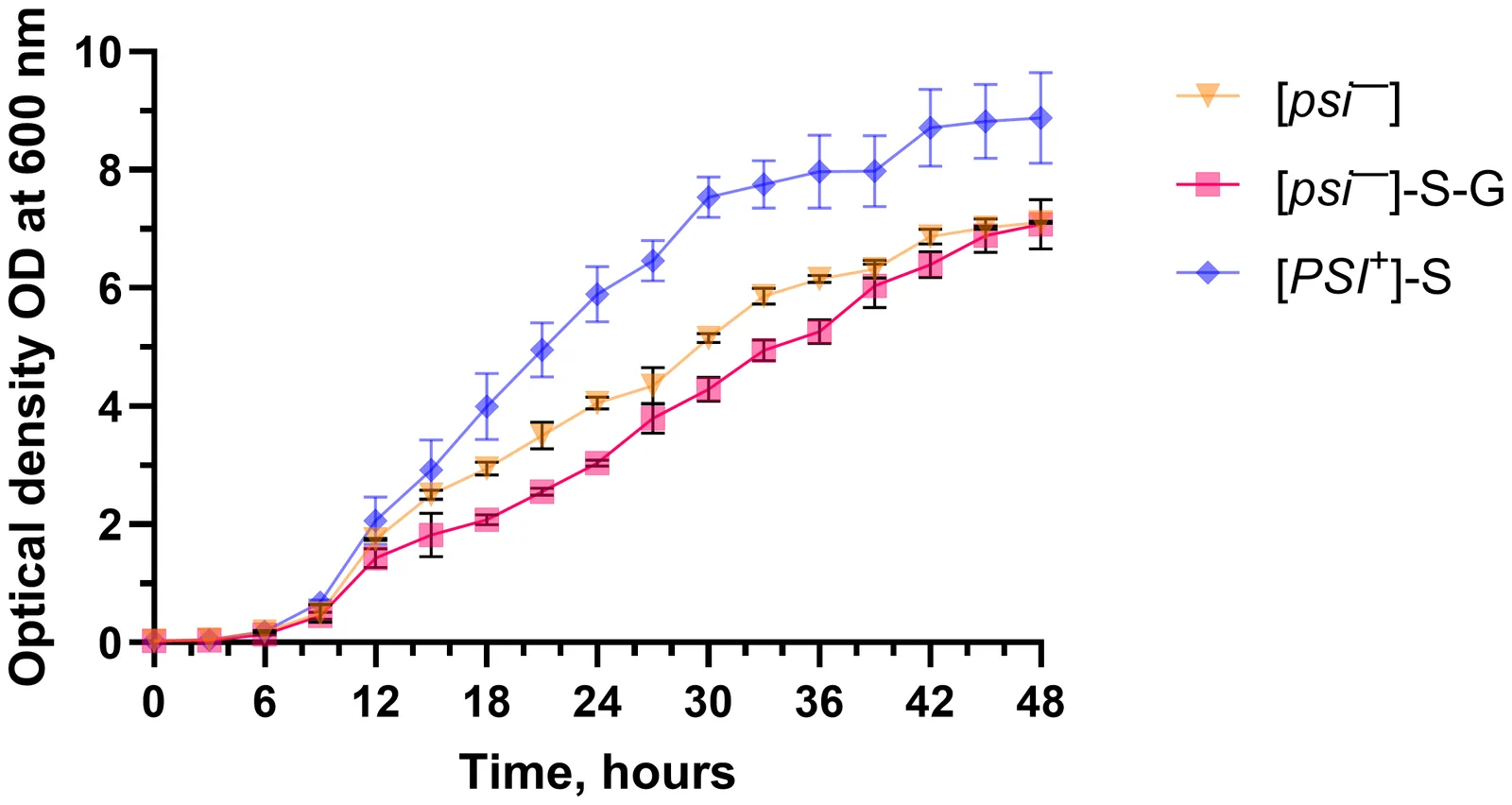
Growth dynamics of the cells with and without [*PSI*^+^] prion (strong variant). OD, optical density. Data presented as average ± SE.

The cells containing a strong [*PSI*^+^] prion variant reached the highest optical density (8.88 ± 0.77 at 48 h). Neither cured cells nor [*psi*^−^] cells reached this level of biomass accumulation. Among the cured strains, cells in which the strong [*PSI*^+^] variant was eliminated using GuHCl alone reached 7.08 ± 0.42 at 48 h, which did not differ significantly from the OD reached by [*psi*^−^] cells that never contained [*PSI*^+^] (7.11 ± 0.01 at 48 h; *p* > 0.05, Table S2.10)

For both weak and strong prion variants, the [*PSI*^+^] cells consistently reached higher OD values than their corresponding cured derivatives and [*psi*^−^] controls. Notably, curing with GuHCl alone allowed cells to reach OD values similar to those of untreated [*psi*^−^] cells, whereas curing using the combined GuHCl + PEF treatment reduced cell reproduction, particularly in the case of the weak variant.

An additional study was performed, in which the [*psi*^−^] cells were exposed to both elimination methods. The highest OD was observed in cells that were not exposed to any of the elimination methods (7.11 ± 0.01 at 48 h; Fig. 6, Tables S2.6–S2.8), indicating that the treatments impact growth independently of prion state.

**Figure 6:**
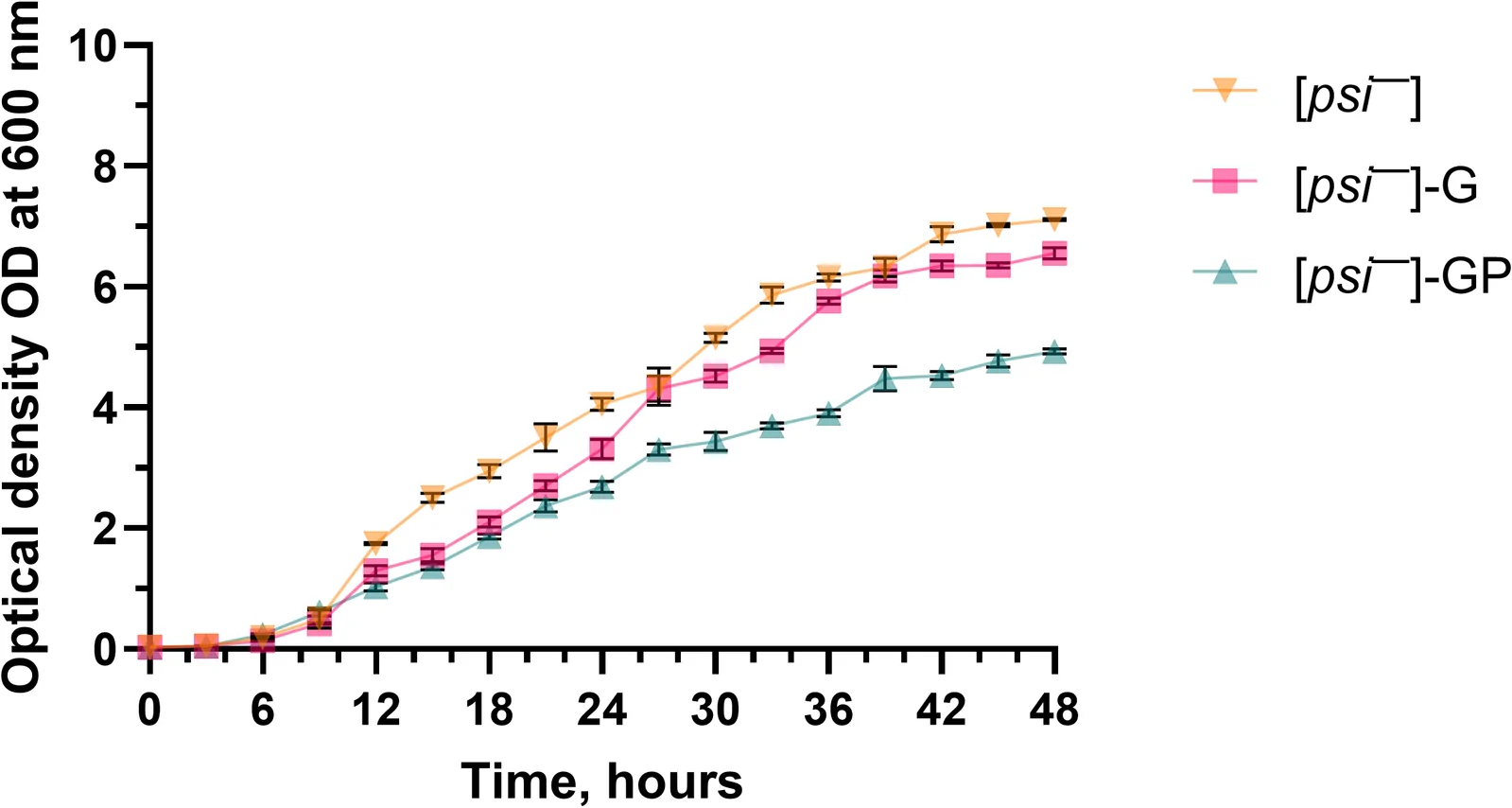
Growth dynamics of the cells without [*PSI*^+^] prion and affected by different elimination methods. OD, optical density. Data presented as average ± SE.

Neither the cells treated with GuHCl alone nor the cells treated with the combined GuHCl + PEF were able to restore cell growth rate of untreated [*psi*^−^]. These differences were statistically significant (*p* < 0.05; Table S2.10). Cells treated with GuHCl alone reached a maximum OD of 6.56 ± 0.1 at 48 h, while cells exposed to the combined GuHCl + PEF treatment reached a maximum of 4.93 ± 0.05 at 48 h. Relative to untreated [*psi*^−^] cells, GuHCl treatment reduced the maximum OD by approximately 1.08-fold, while the combined GuHCl + PEF treatment reduced the maximum OD by approximately 1.44-fold.

### Cell doubling time

Cell doubling time is the time required for a cell population to double in size. Doubling times were calculated from the exponential portions of the growth curves presented above. The doubling time of cells that have never contained the [*PSI*^+^] prion was 1.83 ± 0.07 h (Figs. 7A–7C, represented as a red solid bar; Tables S3.1–S3.8).

**Figure 7:**
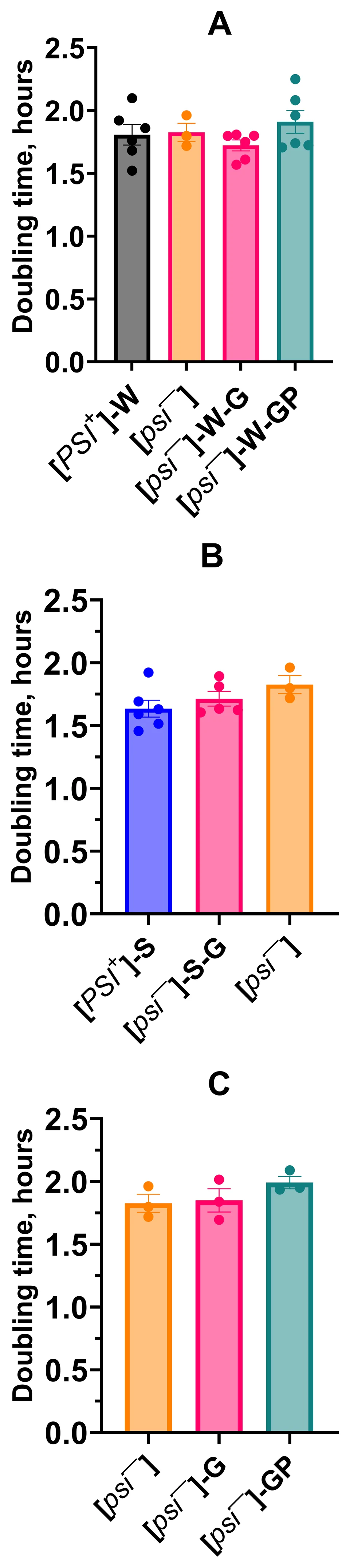
Doubling time of cells with and without [*PSI*^+^] prions under different elimination methods. Data presented as average ± SE. (A) Doubling time of the cells with and without [*PSI*^+^] prion (weak variant). (B) Doubling time of the cells with and without [*PSI*^+^] prion (strong variant). (C) Doubling time of the cells without [*PSI*^+^] prion and affected by different elimination methods.

Cells containing the weak [*PSI*^+^] prion doubled in 1.81 ± 0.08 h, similar to the doubling time of [*psi*^−^]. Cells cured of the weak [*PSI*^+^] prion doubled in 1.72 ± 0.04 h (GuHCl treatment) and 1.91 ± 0.09 h (GuHCl + PEF treatment) (Fig. 7A). Cells cured with GuHCl alone showed a significantly shorter doubling time than those cured with the combined GuHCl + PEF method (*p* < 0.05; Table S3.9). In contrast, no statistically significant differences were found when comparing [*psi*^−^] cells with cells cured of the weak variant by either method (*p* >  0.05, Table S3.10), indicating that loss of the weak [*PSI*^+^] prion restores doubling time to the [*psi*^−^] baseline.

Cells containing the strong [*PSI*^+^] variant doubled in 1.63 ± 0.07 h, whereas cells in which the strong [*PSI*^+^] variant was eliminated using GuHCl alone doubled in 1.71 ± 0.06 h (Fig. 7B). This difference was not statistically significant (*p* > 0.05, Table S3.10), demonstrating that curing the strong variant by GuHCl does not alter doubling time relative to [*psi*^−^] cells.

The doubling times of cells that never contained [*PSI*^+^] prion but were exposed to elimination treatments were 1.85 ± 0.09 h (GuHCl) and 1.99 ± 0.05 h (GuHCl + PEF) (Fig. 7C). Although treated [*psi*^−^] cells showed slightly longer doubling time compared to untreated [*psi*^−^] controls, these differences were not statistically significant (*p* > 0.05, Table S3.10). Thus, the elimination methods affected biomass accumulation (OD) but did not significantly alter exponential phase doubling time.

## Discussion

*S. cerevisiae* is a widely used model for discovering anti-prion compounds and elucidating their mechanisms of action. As a tractable system for modelling protein aggregation, it facilitates studies relevant to amyloid and prion diseases in higher organisms. The long-studied yeast prion [*PSI*^+^] is especially useful for screening anti-prion compounds (Chernova, Chernoff & Wilkinson, 2019). The red/white colony-color assay enables phenotypic discrimination between [*psi*^−^] and [*PSI*^+^] cells and, can distinguish weak and strong [*PSI*^+^] variants particularly when combined with additional phenotypic and biochemical validation (Liebman & Chernoff, 2012). Building on this assay framework, we compared chemical and physical curing strategies and evaluated their effects on exponential-phase growth and maximal biomass accumulation, aspects less explored in previous work.

Selected weak and strong [*PSI*^+^] prion variants were subjected to curing with the classical agent GuHCl and to a combined GuHCl + PEF regimen. PEF can influence cells by transiently increasing membrane permeability, which may facilitate GuHCl uptake (Weaver, 1993), and/or by perturbing non-covalent electrostatic interactions within protein assemblies, potentially remodeling [*PSI*^+^] prion aggregates (Guest, Cashman & Plotkin, 2010). *In vitro*, PEF has been shown to shorten/remodel Sup35NM fibrils (Jurgelevičiūtė et al., 2021). In our experiments with live yeast cells, the combined treatment did not improve the curing of the weak variant and failed to eliminate the strong variant. This outcome may be related to the structural differences between [*PSI*^+^] variants: biophysical studies show variant-specific fibril architectures (*e.g.*, thinner fibrils in strong and thicker in weak) that correlate with distinct fragmentation and propagation behaviors (Ghosh et al., 2018). PEF-induced fibril shortening could, in principle, preserve or increase the number of aggregation-competent particles rather than promote aggregate clearance. However, propagon number and seeding activity were not measured in this study, so this interpretation remains hypothetical.

In this study, we asked whether early growth behavior changes after [*PSI*^+^] removal. To address this, we quantified growth dynamics by (i) calculating doubling time during the exponential growth phase and (ii) measuring the maximal optical density reached in the early stationary phase. This design allowed us to separate effects on growth rate from effects on biomass accumulation, and to compare outcomes across prion states and curing methods

The doubling-time analysis showed that treating [*psi*^−^] cells with GuHCl or the GuHCl + PEF regimen did not alter the exponential growth rate relative to untreated controls (*p* > 0.05). By contrast, in the cured weak [*PSI*^+^] prion background, the method mattered: GuHCl only curing yielded a significantly shorter doubling time than GuHCl + PEF (*p* < 0.05). These findings suggest that the curing method can influence the exponential phase growth rate after prion removal. However, this result should be interpreted cautiously because of the limited sample size, the use of a single yeast genetic background, and the evaluation of only one set of PEF parameters.

In yeast, PEF is known to cause transient electroporation and cell wall remodeling (Ganeva, Galutzov & Teissie, 2014), and at certain field regimes can induce autolysis and loss of viability (Gášková et al., 1996; Martínez et al., 2018); These reported effects provide a possible explanation for the growth pattern observed after GuHCl + PEF treatment. However, viability, membrane integrity, cellular stress responses, and metabolic activity were not measured in this study. Therefore, PEF-induced sublethal damage cannot be identified as the cause of the observed growth differences. Further experiments using viability assays, membrane-integrity measurements, and markers of cellular stress or metabolic activity would be required to test this interpretation.

GuHCl inhibits Hsp104-dependent prion seed replication (Jung & Masison, 2001), whereas PEF-induced shortening of Sup35 fibrils has been demonstrated *in vitro* (Jurgelevičiūtė et al., 2021). Because propagon number and seeding activity were not measured after PEF treatment, the contribution of fibril remodeling to the variant-dependent curing outcome remains hypothetical. Evaluation of maximal optical density showed that, when GuHCl alone was used as the elimination method, cells cured of the [*PSI*^+^] prion reached maximal OD similar to that of [*psi*^−^] cells that had never contained [*PSI*^+^] prion. By contrast, weak [*PSI*^+^]-cured cells treated with the combined GuHCl + PEF method reached a lower maximal OD than [*psi*^−^] controls. To distinguish whether this reflected the elimination method itself or the consequences of prior [*PSI*^+^] presence, we exposed [*psi*^−^] cells (which never contained [*PSI*^+^]) to both treatments. Neither GuHCl-treated nor GuHCl + PEF treated [*psi*^−^] cells reached the OD of untreated [*psi*^−^] cultures (*p* < 0.05), indicating that the treatments can reduce maximal biomass accumulation independently of prion state. The larger reduction observed after the combined treatment is consistent with a treatment-related effect, although the present experiments do not identify the responsible cellular mechanism. Reported effects of PEF on yeast membranes, cell-wall properties, and viability provide relevant context but do not demonstrate that these processes caused the OD differences observed here (Gášková et al., 1996; Ganeva, Galutzov & Teissie, 2014; Jurgelevičiūtė et al., 2021). In contrast, cells that had contained [*PSI*^+^] and were cured with GuHCl alone recovered the same OD as untreated [*psi*^−^] cells for both weak and strong variants. This observation describes the growth outcome under the conditions tested but does not establish that GuHCl caused no other persistent physiological effects.

The question of why cells in which the [*PSI*^+^] prion has been eliminated can nonetheless avoid the full impact of elimination treatments and reach the same maximal optical density as untreated [*psi*^−^] cells remains unclear. The observed growth pattern is associated with the prion and curing history of the strains, but the present experiments do not establish that prior [*PSI*^+^] carriage caused persistent or epigenetic physiological changes. Alternative explanations, including treatment-related effects, clonal variation introduced during isolate selection, and other unmeasured differences among the strains, cannot be excluded. It is well established that [*PSI*^+^] dependent suppression of STOP codons leads to the synthesis of altered proteins: translation continues beyond normal termination sites, producing extended polypeptides that may be unstable, dysfunctional, or functionally altered. When such aberrant proteins accumulate, any hypothetical advantage of the [*PSI*^+^] phenotype can be lost (Wickner et al., 2010; Tuite, 2013). However, several studies have also shown that [*PSI*^+^] can be beneficial under specific conditions, such as unmasking cryptic genetic variation, modulating stress resistance, or prolonging chronological lifespan (Eaglestone, 1999; True & Lindquist, 2000; True, Berlin & Lindquist, 2004; Wang, Melki & Kabani, 2017). In this context, the similar maximal OD reached by GuHCl-cured and untreated [*psi*^−^] cells raises the possibility that prior prion history is associated with subsequent growth behavior, but it does not demonstrate a persistent or advantageous physiological imprint. The cellular basis of the growth differences observed here was not examined directly. Further studies using independently derived isolates and direct measurements of translation, stress responses, and metabolic activity would be required to determine whether prior [*PSI*^+^] carriage contributes causally to post-curing growth behavior.

## Conclusions

In summary, this study shows that adding PEF to GuHCl does not increase [*PSI*^+^] prion elimination efficiency and, in the case of the strong [*PSI*^+^] variant, abolishes curing, whereas GuHCl alone remains effective for both weak and strong variants. At the level of growth phenotypes, PEF-based curing of the weak [*PSI*^+^] background modestly slows exponential phase doubling and reduces the maximal OD reached by [*psi*^−^] cells in early stationary phase, while cells cured of [*PSI*^+^] with GuHCl alone recover growth rates and ODs comparable to [*psi*^−^] controls. Together, these findings indicate that the curing treatment is associated with subsequent growth differences. However, the present study does not establish that prior [*PSI*^+^] carriage causes persistent, epigenetic, or beneficial physiological effects after curing, nor does it identify the cellular mechanisms responsible for the observed growth patterns. These findings should be interpreted cautiously because of the limited sample size, the use of a single yeast genetic background, and the absence of direct measurements of viability, membrane integrity, cellular stress, and metabolic activity.

## Supplemental Information

10.7717/peerj.21775/supp-1 Supplemental Information 1 *S. cerevisiae* 74-D694 colonies grown on SC-Ade media for 21 days Numbers 1–3 indicate independent experimental repeats.

10.7717/peerj.21775/supp-2 Supplemental Information 2 *S. cerevisiae* 74-D694 colonies grown on 1/4YPD media after elimination of strong [PSI^+^] prion variant Prion eliminated using GuHCl alone (1) and combination of GuHCl and PEF (2).

10.7717/peerj.21775/supp-3 Supplemental Information 3 Uncropped blot images from Fig. 2

10.7717/peerj.21775/supp-4 Supplemental Information 4 Raw data in machine readable format

## Additional Information and Declarations

### Data Availability

The following information was supplied regarding data availability: The raw data available in the Supplemental Files.
