# Supplementary material for: Pulsed electric field-mediated elimination of yeast [PSI+] prion is variant dependent and affects cell growth dynamics
Source: PeerJ. 2026 Oct 23;14:e21775. doi: 10.7717/peerj.21775 (PMC13702038; doi:10.7717/peerj.21775)

SDD-AGE experiment. Blot was probed for SUP35 (Abnova #PAB15578). Merged is combined image of whitelight image (for colorimetric ladder) and chemiluminescence, automatically produced by imager. Blue box indicates lanes used for cropped images in Figure 3a.
Samples are the lysates of 74D-694 strains, with the following prion variants: 1 – *psi*$^{-}$, 2 – PSI+-s, 3 – *PSI*$^{+}$-w, 4 – *psi*$^{-}$-w-G, 5 - *psi*$^{-}$-w-GP, *psi*$^{-}$-s-G, *PSI*$^{+}$-s-GP.

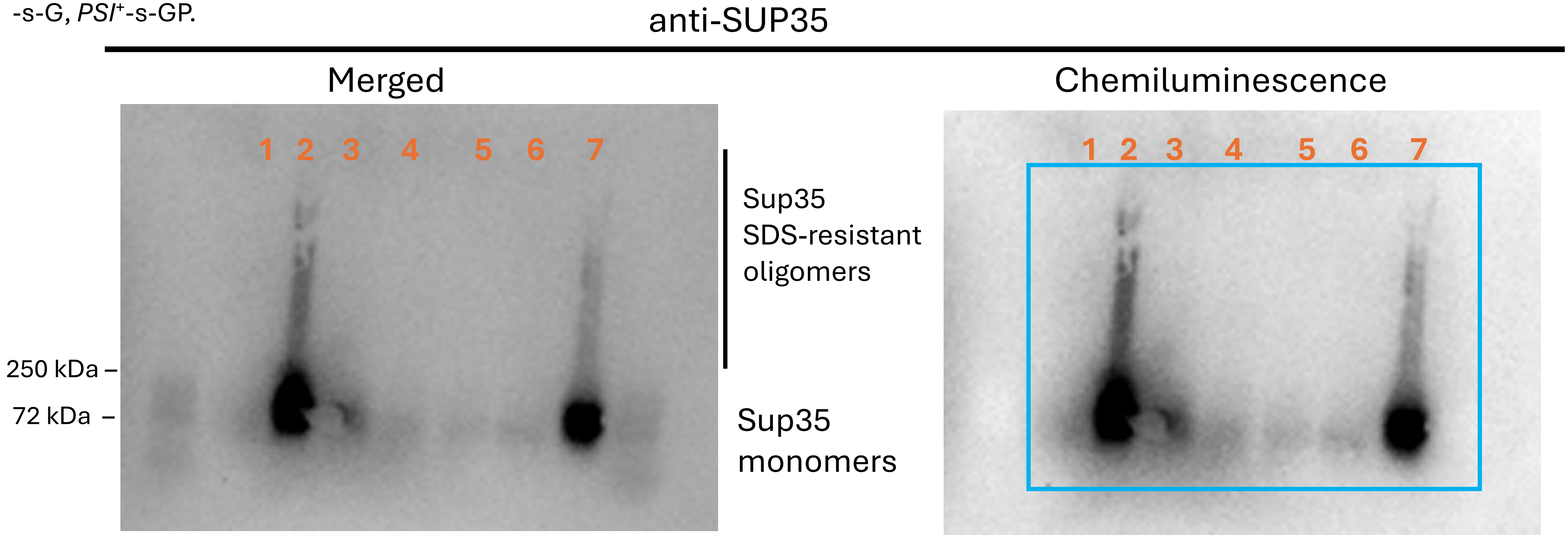

SDS-PAGE experiment. Blot was first probed for SUP35 (Abnova #PAB15578), then stripped, reblocked and probed for GAPDH (Proteintech #HRP-60004). Merged is combined image of whitelight image (for colorimetric ladder) and chemiluminescence, automatically produced by imager. Blue box indicates lanes used for cropped images in Figure 3b.
Samples are the same lysates of 74D-694 strains as in Figure 3a, with the following prion variants: 1 – *psi*$^{-}$, 2 – PSI+-s, 3 – *PSI*$^{+}$-w, 4 – *psi*$^{-}$-w-G, 5 - *psi*$^{-}$-w-GP, *psi*$^{-}$-s-G, *PSI*$^{+}$-s-GP.

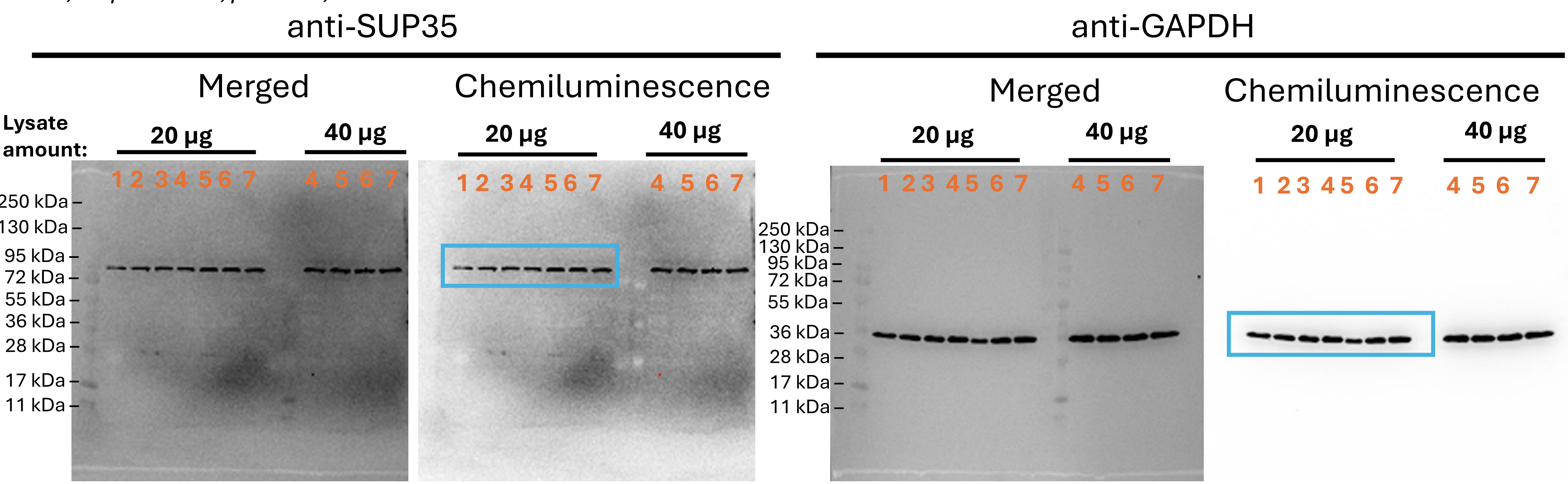

Supplement: Supplementary file 1 [file peerj-14-21775-s003.pdf]
